# Inference of Bacterial Small RNA Regulatory Networks and Integration with Transcription Factor-Driven Regulatory Networks

**DOI:** 10.1128/mSystems.00057-20

**Published:** 2020-06-02

**Authors:** Mario L. Arrieta-Ortiz, Christoph Hafemeister, Bentley Shuster, Nitin S. Baliga, Richard Bonneau, Patrick Eichenberger

**Affiliations:** a Center for Genomics and Systems Biology, Department of Biology, New York University, New York, New York, USA; b Institute for Systems Biology, Seattle, Washington, USA; c Center for Computational Biology, Flatiron Institute, New York, New York, USA; d Center for Data Science, New York University, New York, New York, USA; European Molecular Biology Laboratory

**Keywords:** gene networks, global regulation, small RNAs

## Abstract

Small noncoding RNAs (sRNAs) are key regulators of bacterial gene expression. Through complementary base pairing, sRNAs affect mRNA stability and translation efficiency. Here, we describe a network inference approach designed to identify sRNA-mediated regulation of transcript levels. We use existing transcriptional data sets and prior knowledge to infer sRNA regulons using our network inference tool, the *Inferelator*. This approach produces genome-wide gene regulatory networks that include contributions by both transcription factors and sRNAs. We show the benefits of estimating and incorporating sRNA activities into network inference pipelines using available experimental data. We also demonstrate how these estimated sRNA regulatory activities can be mined to identify the experimental conditions where sRNAs are most active. We uncover 45 novel experimentally supported sRNA-mRNA interactions in Escherichia coli, outperforming previous network-based efforts. Additionally, our pipeline complements sequence-based sRNA-mRNA interaction prediction methods by adding a data-driven filtering step. Finally, we show the general applicability of our approach by identifying 24 novel, experimentally supported, sRNA-mRNA interactions in Pseudomonas aeruginosa, Staphylococcus aureus, and Bacillus subtilis. Overall, our strategy generates novel insights into the functional context of sRNA regulation in multiple bacterial species.

**IMPORTANCE** Individual bacterial genomes can have dozens of small noncoding RNAs with largely unexplored regulatory functions. Although bacterial sRNAs influence a wide range of biological processes, including antibiotic resistance and pathogenicity, our current understanding of sRNA-mediated regulation is far from complete. Most of the available information is restricted to a few well-studied bacterial species; and even in those species, only partial sets of sRNA targets have been characterized in detail. To close this information gap, we developed a computational strategy that takes advantage of available transcriptional data and knowledge about validated and putative sRNA-mRNA interactions for inferring expanded sRNA regulons. Our approach facilitates the identification of experimentally supported novel interactions while filtering out false-positive results. Due to its data-driven nature, our method prioritizes biologically relevant interactions among lists of candidate sRNA-target pairs predicted *in silico* from sequence analysis or derived from sRNA-mRNA binding experiments.

## INTRODUCTION

Small noncoding RNAs (sRNAs) are key regulators of global bacterial gene expression (1–5). Via complementary base pairing to their mRNA targets, sRNAs modulate recognition of transcripts by complexes such as ribosomes and ribonucleases (3). sRNAs can be classified as either *trans*-encoded (when they regulate genes regardless of their chromosomal location) or *cis*-encoded (when they regulate adjacent genes only) (3, 6). Here, we focus on *trans*-encoded sRNAs affecting mRNA stability. Because transcription factors (TFs) and sRNAs share targets or even regulate each other (7), a comprehensive characterization of any gene regulation network must incorporate both types of regulators (8).

Escherichia coli is currently the bacterial species with the highest number of well-characterized sRNA-mRNA interactions, where 22 sRNAs and their targets form an intricate network of 102 experimentally supported interactions (9); however, this number is likely a fraction of sRNA-mediated regulation in that species (10, 11). Accurate and comprehensive detection of sRNA-mRNA interactions is challenging, experimentally and computationally. Experimental strategies are complicated by the difficulty of identifying the conditions in which sRNAs are active (12). Computational methods predicting sRNA-mRNA interactions are fast and inexpensive, but they have a high false-positive rate and often fail to recall known targets (5, 9).

Network inference methods have been implemented to study sRNA-mediated regulation and map E. coli sRNA regulatory networks using transcriptional profiles of sRNAs and their putative target genes. These strategies rely either on context likelihood of relatedness (CLR) or exploit gene coregulation; however, the recall of known sRNA-mRNA interactions is limited, and the accuracy of novel predictions is not systematically evaluated (13–15). Contrary to what was assumed in previous network inference strategies, sRNA levels might not be an adequate proxy for their regulatory activity. Confounding factors include RNA chaperones (such as Hfq) that promote sRNA-mRNA interactions (4, 5, 16) and ribonucleases that are required to activate some sRNAs by processing (e.g., RNase Y-mediated modification of RoxS in Bacillus subtilis) (17). Moreover, the regulatory contribution of sRNAs becomes inconsequential when the concentration of their targets significantly exceeds their own concentration (18, 19).

In this work, we address the complexity of sRNA-mediated regulation by estimating sRNA regulatory activities to generate models of gene regulation for four bacterial species. We show that our pipeline outperforms previous efforts, detects novel sRNA-mRNA interactions, and complements RNA-RNA interaction prediction methods by discriminating between true and false targets. By identifying the most likely sRNA targets, our strategy can help researchers to select promising interactions for experimental validation.

## RESULTS AND DISCUSSION

We inferred sRNA regulons from transcriptomic data using either the *Inferelator* (20) or CLR (13, 21). A set of experimentally supported sRNA-mRNA interactions (referred to as sRNA priors; see Table S1 in the supplemental material) was used for estimating sRNA regulatory activities (SRAs). Because it mines transcriptomic data, our approach is designed to identify sRNA-mRNA interactions that change mRNA stability. The initial analysis was restricted to eight E. coli sRNAs with several experimentally supported targets (Table 1) and then expanded to B. subtilis, Pseudomonas aeruginosa, and Staphylococcus aureus. The accuracy of the inferred sRNA regulons was assessed by analysis of publicly available experimental data.

**TABLE 1:** Escherichia coli sRNAs analyzed in this study

sRNA	Biological process	Prior target genes	No. of candidate target genes^*a*^	Reference(s)
CyaR	Sugar metabolism^*b*^	*luxS*, *nadE*, *ompX*, *ptsI*, *yobF*, *yqaE*	28	29, 75
FnrS	Anaerobic respiration^*b*^	*cydD*, *folE*, *folX*, *gpmA*, *maeA*, *marA*, *metE*, *sodA*, *sodB*, *yobA*	59	29, 72, 73
GcvB	Amino acid metabolism and transport^*c*^	*argT*, *csgD*, *cycA*, *gdhA*, *livJ*, *lrp*, *phoP*, *sstT*, *yifK*	87^*d*^	34, 76–79
MicA	Stress response^*b*^	*ecnB*, *fimB*, *lamB*, *lpxT*, *ompA*, *ompW*, *ompX*, *tsx*, *ycfS*, *yfeK*	15	80
OmrA/OmrB^*e*^	Stress response (membrane)^*b*^	*cirA*, *csgD*, *fecA*, *fepA*, *ompR*, *ompT*	46	81, 82
RybB	Stress response^*b*^	*fadL*, *fiu*, *lamB*, *nmpC*, *ompA*, *ompC*, *ompF*, *ompW*, *rluD*, *tsx*	22	80
RyhB	Iron metabolism^*b*^	*acnA*, *cysE*, *dmsA*, *erpA*, *fumA*, *fumB*, *msrB*, *nagZ*, *sodB*, *uof*, *ykgJ*, *ynfF*	84^*f*^	28, 29, 31
Spot 42	Sugar metabolism and transport^*c*^	*ascF*, *fucI*, *galK*, *glpF*, *gltA*, *maeA*, *nanC*, *paaK*, *puuE*, *srlA*, *sthA*, *xylF*	42^*g*^	35, 83

a Potential targets were extracted from studies reporting transcriptional profiling data and validated targets.

b Refers to the process that influences the expression of the corresponding sRNA.

c The sRNA controls multiple targets associated with the indicated biological processes.

d Includes targets detected in *Salmonella* Typhimurium.

e OmrA and OmrB were considered a single regulator (OmrA) in the analyses.

f Includes ribosome profiling data. Targets labeled as indirect RyhB targets in Wang et al. (28) were not considered.

g Includes differentially expressed genes detected with our reanalysis, using Cyber-T (37), of transcriptomic data reported in reference 35.

10.1128/mSystems.00057-20.3 TABLE S1 Manually selected sRNA-mRNA interactions used as sRNA priors in this study. Download Table S1, DOCX file, 0.02 MB. Copyright © 2020 Arrieta-Ortiz et al. 2020 Arrieta-Ortiz et al. This content is distributed under the terms of the Creative Commons Attribution 4.0 International license.

### sRNA transcript level is not a good proxy for regulatory activity in a network inference context.

**FIG 1:**
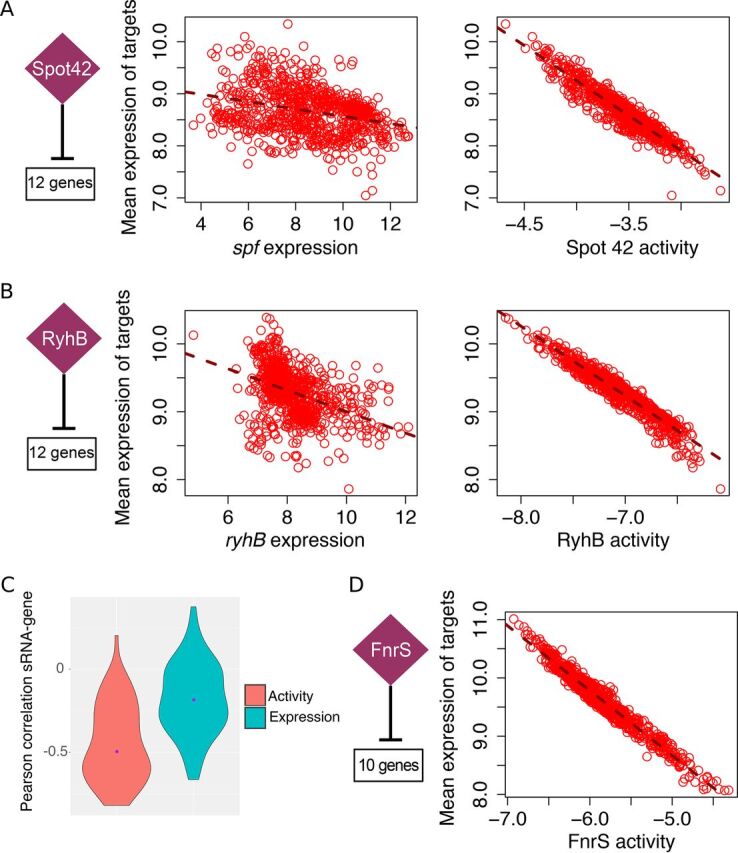
The transcriptional profile of an sRNA is a suboptimal proxy for its regulatory activity. The motivation for estimating sRNA activities is illustrated for three E. coli sRNAs. sRNA activities were estimated for each experimental condition. Each circle represents the value for one microarray experiment. The numbers of known targets used to estimate sRNA activities and to compute the mean expression of the analyzed regulons (under each condition) are indicated. (A) Spot 42 controls the uptake and metabolism of alternative sugars (35). A stronger relation is observed between the estimated Spot 42 activity and the mean expression profile of its dependent genes (right panel) than between the expression profile of *spf* and its targets (left panel). (B) RyhB represses production of iron-consuming proteins as part of the iron-sparing response (28, 31). Similarly, the relation between estimated RyhB activity and the mean expression profile of its targets is stronger than the relationship between the expression profile of *ryhB* and its targets. (C) Violin plots show the distribution of Pearson correlation values between sRNAs and the transcriptional profile of their priors when either estimated sRNA activities or sRNA transcriptional profiles are used for computation. Purple dots indicate median correlation values (−0.5 and −0.19 for sRNA activity and sRNA transcriptional profiles, respectively). The difference between both sets of correlation values is statistically significant (*t* test *P* value = 9.3e−10). (D) FnrS is associated with anaerobic respiration (72, 73). The probes for *fnrS* did not need to be present in the E. coli transcriptomic data set in order to be included as a potential regulator in our pipeline. FnrS activity was estimated from the expression profile of 10 FnrS-dependent genes present in the transcriptomic compendium (see Table S1 in the supplemental material).

Transcriptional profiles of regulators (i.e., expression levels in a given transcriptomic compendium) commonly serve as proxies for their regulatory activity (14, 15), even though the outcome of sRNA-mediated regulation is influenced by RNA chaperones, ribonucleases, RNA sponges, and target mRNA concentrations. It was also previously demonstrated that the activity of TFs does not consistently match their transcription profiles (22, 23). Figure 1A and B and Fig. S1 in the supplemental material display the transcriptional profiles of several sRNAs against the average transcriptional profile of their experimentally supported targets. In E. coli and other species (e.g., B. subtilis and S. aureus), sRNA transcript levels exhibited only a weak linear relation with their mRNA targets, implying that sRNA transcript levels are less than optimal proxies for sRNA regulatory activity in network inference procedures.

10.1128/mSystems.00057-20.1 FIG S1 Motivation for estimating the regulatory activity of sRNAs in bacteria. sRNA activities were estimated for each experimental condition. Each dot represents one microarray experiment. The number of experimentally supported targets used to estimate sRNA activities and to compute the mean expression of the analyzed regulons (under each condition) is indicated. In all cases, a stronger relation is observed between the estimated sRNA activities and the average expression profile of their dependent genes (right panels) than between the expression profile of the sRNAs and the average expression profile of their targets (left panels). (A) E. coli CyaR is expressed when levels of cyclic AMP (cAMP) are high (75). (B) E. coli GcvB regulates genes involved in amino acid transport and amino acid biosynthesis (32, 76). (C) E. coli MicA is a stress-related sRNA (80). (D) E. coli OmrA is important in the response to membrane stress (81, 82). (E) E. coli RybB is a stress-related sRNA (80). MicA and RybB have multiple targets in common. (F) FsrA is involved in the iron-sparing response of B. subtilis (45). FsrA is a functional analog of E. coli RyhB. (G) RsaE regulates TCA cycle and arginine catabolism in S. aureus (52). Download FIG S1, PDF file, 2.4 MB. Copyright © 2020 Arrieta-Ortiz et al. 2020 Arrieta-Ortiz et al. This content is distributed under the terms of the Creative Commons Attribution 4.0 International license.

### Estimating sRNA regulatory activity.

To estimate SRA, we used transcriptional profiles of experimentally supported sRNA targets. Thus, estimated SRAs can account for multiple mechanisms of sRNA-mediated control of mRNA stability. Conceptually, this is analogous to relying on a reporter gene, except that every presumed target of the sRNA is considered in the estimation, as previously done for TF activity (TFA) estimation (24). We checked the relationship between estimated SRAs and the transcriptional profiles of their respective priors (Fig. 1A and B and Fig. S1). As expected based on work with TFA (24), there was a stronger linear relationship between target genes and the SRA of their corresponding sRNA regulator than with raw sRNA transcript levels. Due to the repressive nature of sRNA-mRNA interactions used as priors, SRA and their targets were usually anticorrelated (Fig. 1C). Nonetheless, positive sRNA-mRNA interactions can also be used as priors and predicted in our approach (similar to TFs that can act both as repressors and activators). Importantly, estimated SRAs can be used for network inference even when the transcriptomic data set does not contain information about the transcription of sRNAs of interest (which happens frequently in microarray-collected data sets). In our workflow, the only requirement for including an sRNA as a potential regulator is the availability of the transcriptional profiles of a set of candidate targets. For instance, despite the absence of FnrS in the transcriptomic data set, its activity was estimated using 10 priors (Fig. 1D).

### General strategy for inferring sRNA regulons.

**FIG 2:**
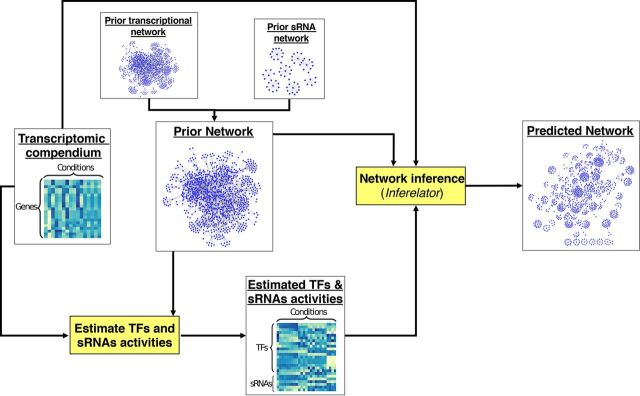
General strategy. A transcriptomic data set and a prior network (built from experimentally supported TF-gene and experimentally supported or candidate sRNA-mRNA interactions) are used for estimating the regulatory activities of TFs (TFAs) and sRNAs (SRAs) using a network component analysis approach (24, 68). Next, estimated TFAs and SRAs, transcriptomic data, and prior network are used as input for the *Inferelator* to infer a regulatory network composed of a transcriptional layer (TF based) and a posttranscriptional layer (sRNA based).

Our network inference pipeline is displayed in Fig. 2. TFAs and SRAs were estimated using a transcriptomic data set (from a public repository such as the Many Microbe Microarrays database) (25) and a set of experimentally supported TF-gene and sRNA-mRNA interactions (prior network) from RegulonDB (26), RegPrecise (27), or equivalent. Next, all of this information was used as inputs to simultaneously infer the TF-controlled and sRNA-controlled networks with the *Inferelator* (20, 24). Predicted interactions not included in the prior network were considered novel. Inclusion of a prior TF network, much larger than the prior sRNA network, was necessary to define thresholds (calibrated using desired precision values) for selecting the interactions that should be kept in the final models. This strategy also prevented overfitting due to an incomplete set of regulators and interactions to explore and enabled us to study connections between the transcriptional and posttranscriptional regulatory layers.

### Our strategy improves performance, recovers known interactions, and predicts novel sRNA-mRNA interactions.

**FIG 3:**
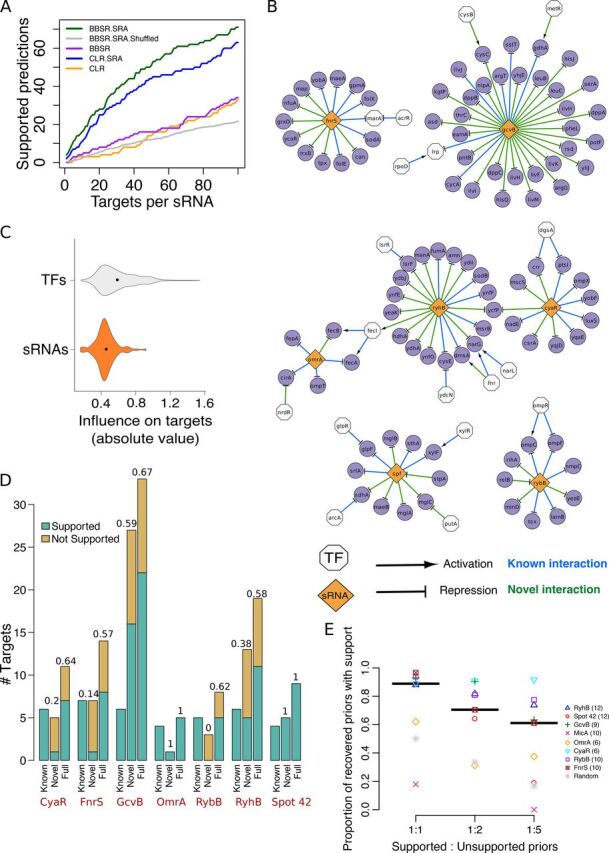
Performance of the *Inferelator* and alternative computational methods for expanding sRNA networks. (A) Performance of the *Inferelator* (BBSR) and mixed CLR, an alternative method, with incorporation of sRNA activities (SRA suffix) and without incorporation of sRNA activities. Genes predicted as targets but not used for sRNA activity estimation were considered to be experimentally supported if they were included in the compiled list of candidate targets of the corresponding sRNA (Table 1). Most candidate targets were differentially expressed in transcriptional profiling experiments (deletion or overexpression of *cyaR*, *gcvB*, *micA*, *omrA*, *spf* [encoding Spot 42], *rybB*, and *ryhB*). Additionally, predicted targets were considered experimentally supported when they were part of an operon containing differentially expressed genes or other validated targets. For each sRNA, targets were ranked based on confidence score (in the case of the *Inferelator*) or mutual information-based score (in the mixed-CLR runs). To estimate the basal performance level of the *Inferelator*, the average of 10 runs with shuffled sRNA priors was also computed (gray line). (B) The inferred sRNA regulatory network of E. coli. To allow comparison between transcriptional and posttranscriptional networks, overlap between both networks is displayed. (C) Violin plots showing the distribution of absolute values of Bayesian regression coefficients (which indicate magnitude) associated with TF-gene and sRNA-mRNA interactions. Black dots indicate the median values. (D) The inferred sRNA regulons are experimentally supported (description of each sRNA regulon in Data Set S1 in the supplemental material). Experimental support rate for novel predictions (not in the prior network) and full inferred regulons (recovered priors and novel predictions) of the BBSR.SRA run shown in panel A are shown above each bar. (E) The *Inferelator* identifies experimentally supported targets among noisy priors. Experimental support rates for recovered priors are plotted for different levels of noise in the priors. Each symbol shows the mean value of 10 *Inferelator* runs (each run with a different set of false priors). Each colored symbol corresponds to one of eight sRNAs. Black lines indicate the median of the average proportions for all eight sRNAs. The gray stars indicate the average expected proportion if priors included in the predicted networks were randomly selected. The numbers of true sRNA targets are shown in parentheses.

We compared the performance of the *Inferelator* (using a Bayesian best subset regression [BBSR]) and a mixed CLR (a modified version of CLR) (21), with and without incorporating SRAs (Fig. 3A). The rank of putative sRNA targets identified with transcriptomic experiments in the list of predicted sRNA targets informs about the performance of the approach (10). Genes used as priors for SRA estimation were removed from the set of predicted targets because they tend to occupy high positions in the prediction ranking. FnrS was not considered because its transcriptional profile was missing from the transcriptomic data set. A predicted target was considered experimentally supported if it was included in the set of candidate targets of its putative regulator (retrieved mainly from transcriptional profiling experiments overexpressing or deleting sRNAs) (Table 1) or when the predicted target was part of an operon that contains differentially expressed genes or other validated targets. For RyhB, ribosome profiling data were also considered (28). Among the 140 predictions (top 20 predictions for seven E. coli sRNAs) made by the *Inferelator* with SRA (BBSR.SRA), 28 were experimentally supported (25 for mixed CLR). In contrast, the *Inferelator* without SRA predicted only eight experimentally supported targets (three for mixed CLR). The BBSR.SRA performed best for Spot 42 (10 supported targets in the top 20) and GcvB (9 supported targets). In general, we observed that incorporation of SRA consistently improved the detection power of both *Inferelator* and CLR (Fig. 3A, green and blue lines [with SRAs] versus purple and orange lines [without SRAs]).

10.1128/mSystems.00057-20.7 DATA SET S1 List of sRNA-mRNA interactions inferred using manually curated sRNA priors for E. coli, P. aeruginosa, B. subtilis, and S. aureus. Download Data Set S1, XLSX file, 0.03 MB. Copyright © 2020 Arrieta-Ortiz et al. 2020 Arrieta-Ortiz et al. This content is distributed under the terms of the Creative Commons Attribution 4.0 International license.

The E. coli sRNA network inferred using BBSR.SRA is shown in Fig. 3B. Limited overlap was observed between the sRNA and TF networks. Only 19% of sRNA-regulated genes were also predicted as targets of one or more TFs. Although 41% of the regulated genes in the prior network had two or more regulators, expression of most genes was explained as the function of a single regulator’s activity. In many cases, the regulatory influence of an sRNA surpassed that of TFs targeting the same gene. For example, *marA* was regulated by five TFs and one sRNA (FnrS) in the prior network. Only the interactions between *marA* and FnrS and one TF (AcrR) were recalled into the final model. Nevertheless, TFs were commonly the most influential regulator, and on average, the influence of sRNAs is subtler than the one exerted by TFs (Fig. 3C), in agreement with previous studies (3). In an alternative *Inferelator* run (where sRNAs were not considered as regulators), 90% of the genes exclusively regulated by sRNAs in our combined network (Fig. 3B) lacked regulatory hypotheses (data not shown), suggesting that inclusion of sRNAs expands the gene regulation models.

The accuracy of inferred sRNA regulons was assessed using previously published studies (see Data Set S1 in the supplemental material). Thirty-eight sRNA-mRNA interactions from the prior network were kept in the final model (total recall of 0.51). The average recall per sRNA regulon was 0.55, and the highest recall was obtained for CyaR (1.0). The inferred sRNA network contained 61 novel interactions, including 29 with experimental support (0.48 support rate) as shown in Fig. 3D. Per regulon, the average experimental support was 0.47 for novel predictions (0.73 when considering both novel predictions and recovered priors). Failure to recall MicA targets is likely a consequence of the weak correlation between estimated activity and the transcription profiles of MicA’s known targets (Fig. S1C). MicA activity may be masked by the action of coregulators (e.g., MicA and RybB share four targets [Table 1]), in agreement with the observation that TFs sharing multiple targets had the lowest recall rate (24). Although we intentionally left out some sRNA targets to estimate the accuracy of our procedure, in future applications, priors’ sets can be expanded by including every differentially expressed gene. In conclusion, integration of estimated SRAs in network inference procedures greatly improves the ability to detect accurate sRNA-mRNA interactions.

### Robustness to incorrect prior information.

In the procedure described above, priors included only experimentally supported sRNA-mRNA interactions; however, in a more realistic scenario, priors would be compiled from heterogenous sources and a mix of true and false interactions is expected. Previously, we showed that the *Inferelator* is robust to noisy priors (up to 1:10 ratio of true/false priors) (24). To confirm this result in the context of sRNAs, we added different amounts of false interactions to the sRNA priors, ran the pipeline with these noisy priors, and found that our method efficiently distinguishes true interactions from false interactions (Fig. 3E). Although the number of recovered priors with experimental support is lower than in the original run without false priors (Fig. S2), the average proportion of recovered priors still exceeded the ratio expected from a random selection (gray stars in Fig. 3E). Thus, this strategy successfully filters out priors not supported by transcriptional data (20, 24).

10.1128/mSystems.00057-20.2 FIG S2 Presence of false sRNA-mRNA interactions reduced the number of sRNA priors included in the networks inferred by the *Inferelator*. Each dot is the mean value of ten *Inferelator* runs (each one with a different set of false priors). Each colored symbol corresponds to one of seven sRNAs with recovered priors. Black lines indicate the medians of the average proportions for all seven sRNAs. The number of true targets for each sRNA is shown in parentheses. (A) Ratio between the number of recovered priors in the *Inferelator* runs with noisy sRNA priors and the total number of recovered priors in the *Inferelator* run without false-positive interactions. (B) Ratio between number of recovered priors with experimental support (true priors) in *Inferelator* runs with noisy sRNA priors and the total number of recovered priors in the *Inferelator* run without false-positive interactions. Download FIG S2, PDF file, 0.02 MB. Copyright © 2020 Arrieta-Ortiz et al. 2020 Arrieta-Ortiz et al. This content is distributed under the terms of the Creative Commons Attribution 4.0 International license.

### Combining sequence-based predictions with transcriptomic data using the *Inferelator*.

**FIG 4:**
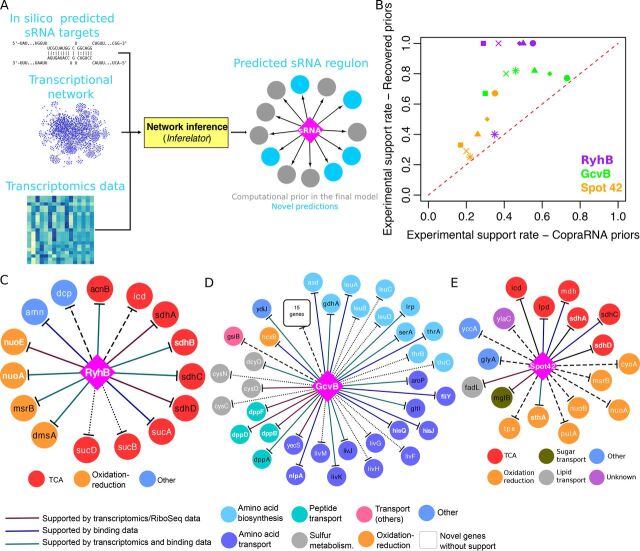
The *Inferelator* identifies computationally predicted sRNA-mRNA interactions with experimental support. (A) General strategy to integrate computational sRNA-mRNA predictions in our pipeline. The resulting sRNA regulons are then analyzed to identify sequence-based sRNA-mRNA interactions supported by available experimental data and potential additions to the sRNA regulon. (B) The experimental support rate of recovered priors is significantly higher than the rate of the original CopraRNA-derived sRNA priors. The six points per sRNA correspond to the six sets of sRNA priors derived from CopraRNA predictions (Table S2). (C) The inferred RyhB regulon when CopraRNA predictions associated with enriched functional terms were used as priors. (D) The inferred GcvB regulon when CopraRNA predictions with *P* values of ≤0.01 were used as priors. Based on the high number of common GcvB targets in E. coli and *S.* Typhimurium, experimental data from *S.* Typhimurium was considered supporting evidence. (E) The inferred Spot 42 regulon when CopraRNA predictions with *P* values of ≤0.01 and associated with enriched functional terms were used as priors. In panels C to E, diamonds and circles represent sRNAs and target genes, respectively. Solid lines indicate interactions with experimental support. Dashed lines indicate interactions without experimental support; dotted lines indicate targets without direct support but located in the same operon of experimentally supported targets. Priors included in the final regulon are shown with black text. Novel targets (i.e., not present in the priors) are shown by white text. Bold white font indicates validated novel targets. Target genes are colored according to their functional annotation (from the EcoCyc database) (74).

We exploited the robustness of the *Inferelator* to noisy priors to separate true positive from false-positive interactions among computationally predicted sRNA-mRNA interactions (Fig. 4A). For any sRNA of interest, we built a set of priors using sequence-based predictions of sRNA-mRNA interactions. Next, we ran the *Inferelator* to recover the most likely pairs. CopraRNA (29), a state-of-the-art RNA-RNA interaction prediction method was employed to produce these priors (9). Standard CopraRNA outputs contain 100 predictions (ranked by the associated *P* values) and likely include many false-positive interactions.

10.1128/mSystems.00057-20.4 TABLE S2 The *Inferelator* filters CopraRNA-derived priors and predicts novel sRNA-mRNA interactions with experimental support. Download Table S2, DOCX file, 0.01 MB. Copyright © 2020 Arrieta-Ortiz et al. 2020 Arrieta-Ortiz et al. This content is distributed under the terms of the Creative Commons Attribution 4.0 International license.

We focused our analysis on the E. coli sRNAs RyhB, GcvB, and Spot 42, since each may directly or indirectly regulate dozens of genes (Table 1). We hypothesized that if we used CopraRNA predictions as priors, our downstream activity estimation and network inference method would further distinguish between true-positive and false-positive interactions and also detect novel interactions. From the compiled sets of candidate sRNA targets (Table 1) and binding data reported by Melamed et al. (10), we estimated that 25% of the CopraRNA predictions were experimentally supported (Table S2). For each sRNA, we ran our pipeline using six sets of priors: (set i) the full set of CopraRNA predictions, (set ii) targets with *P* values of ≤0.01, (set iii) targets associated with enriched functional terms, (set iv) the intersection of sets ii and iii, (set v) the union of sets ii and iii, and (set vi) the union of the top 15 targets based on *P* value (as suggested in the original CopraRNA publication [29]) and set iv. Experimental support rate across the sets ranges from 0.17 to 0.73.

We observed that, in general, running the *Inferelator* dramatically shrank the initial set of priors (Table S2), while the experimental support rate increased significantly (Fig. 4B). We found that 22 out of the 43 (51.2%) CopraRNA-derived priors included in the final networks had been independently validated (Data Set S2). Nine additional sRNA recalled priors are experimentally supported (but not validated yet). Regarding novel predictions, out of 159 interactions, 18 were independently validated, and 33 were experimentally supported (Data Set S2). A subset of 34 predicted interactions supported by experimental data (physical binding, transcriptional profiling, and validation in a closely related species such as Salmonella enterica serotype Typhimurium) are listed in Table 2. For example, the RyhB-*cheY* interaction is supported in E. coli by physical binding data and significant upregulation of *cheY* in an *S.* Typhimurium strain missing one of its two RyhB genes (10, 30). Similarly, the RyhB-*mrp* interaction is supported by downregulation of *mrp* when RyhB is overexpressed (31), physical interaction (10), and increased translation rate of *mrp* in a *ryhB* deletion strain (28).

**TABLE 2:** Experimentally supported new members of the RyhB, GcvB, and Spot 42 regulons identified using CopraRNA-derived sRNA priors^*a*^

sRNA	Target gene	Exptl support^*b*^	Recovered prior?^*c*^	Prior set(s)^*d*^	Reference(s)
RyhB	*acpP*	B	No	ii	10
RyhB	*amn*	B	No	iii, v	70
RyhB	*cheY*	B, S	Yes	i	10, 30
RyhB	*fabZ*	B	No	vi	10, 70
RyhB	*folX*	B	No	ii	10
RyhB	*gshB*	B	No	ii	10
RyhB	*mrp*	TD, B, RP	No	iv	10, 28, 31, 70
RyhB	*rna*	B	No	ii	70
RyhB	*rsmE*	B	No	ii, vi	70
RyhB	*tpx*	B	No	i	10
RyhB	*ubiD*	B	No	ii, vi	10
RyhB	*ybaB*	B	No	ii, iv, vi	10
GcvB	*aroG*	B	No	v	32
GcvB	*aroP*	B, S	Yes	ii−vi	10, 32, 34
GcvB	*asd*	B	No	ii−vi	10
GcvB	*cysD*	TD	No	ii, v	76
GcvB	*dcyD* (*yedO*)	B, S	No	i, ii, iv−vi	10, 34
GcvB	*hcxB* (*ybiC*)	B	No	ii−vi	10, 32
GcvB	*icd*	B, S	No	i	10, 84
GcvB	*ilvN*	TD, S	No	iii, iv, vi	34, 76
GcvB	*leuA*	B	No	ii−vi	32
GcvB	*purU*	B, S	No	i	10, 32, 34
GcvB	*ydiJ*	B, S	Yes	ii, v	10, 32, 34
GcvB	*yecS*	S	No	ii	34
Spot 42	*fabA*	TD^*e*^	No	i	35
Spot 42	*fadL*	TD^*e*^	Yes	iv	35
Spot 42	*lpd*	B	Yes	iv, vi	10
Spot 42	*lysS*	B, I	Yes	i	10, 85
Spot 42	*mdh*	B	No	iv, vi	10
Spot 42	*mglB*	TD^*e*^, S	Yes	iii, iv	35, 39
Spot 42	*rbsB*	TD^*e*^	No	iii	35
Spot 42	*tktA*	B	Yes	i	10
Spot 42	*yjiA*	B, TD	Yes	v	10, 35
Spot 42	*yjjK*	TD^*e*^, I	No	i, ii, v, vi	35, 85

a A complete description of experimental data that support listed interactions is available in Data Set S2.

b Experimental support is indicated as follows: B, support from physical sRNA-mRNA interaction data; S, support from studies with *S*. Typhimurium; TD, support from transcriptional data; RP, support from ribosome profiling data; I, indirect support (e.g., differential expression in *hfq* deletion strain).

c Predicted targets were either sRNA priors (included in the CopraRNA predictions used to infer sRNA regulons) or novel targets.

d Refers to the six versions of CopraRNA-derived sRNA priors described in Results and Discussion.

e Gene was identified as differentially expressed in our reanalysis of the transcriptional data reported in reference 35.

10.1128/mSystems.00057-20.8 DATA SET S2 List of sRNA-mRNA interactions inferred using CopraRNA-derived priors for RyhB, GcvB, and Spot 42. Download Data Set S2, XLSX file, 0.03 MB. Copyright © 2020 Arrieta-Ortiz et al. 2020 Arrieta-Ortiz et al. This content is distributed under the terms of the Creative Commons Attribution 4.0 International license.

For RyhB, best results were obtained with a set of priors containing 38 genes associated with enriched functional terms (Fig. 4C). The inferred RyhB regulon had a 0.73 accuracy (11 experimentally supported targets out of 15). In addition to six recalled validated priors, nine additional targets were predicted (five of which had experimental support [Data Set S2]). Genes involved in respiration (*nuoA* and *nuoE*), the tricarboxylic acid (TCA) cycle (*sucA-sucB-sucD*), and nucleoside metabolism (*amn*) were among the novel predictions.

Even when using different prior sets, large regulons were systematically inferred for GcvB (Table S2), in line with its global regulatory role (32). Figure 4D displays the inferred GcvB regulon for CopraRNA predictions with *P* values of ≤0.01. Eleven priors (out of 46) were recalled. Ten of these 11 priors were previously validated or experimentally supported (Data Set S2). Thirty-nine genes were predicted as novel targets, including seven validated targets (*fliY*, *hisJ*, *hisQ*, *dppB*, *dppD*, *dppF*, and *nlpA*). Among these, *hisJ* and *hisQ* are in the same operon as *argT*, a known GcvB target. Similarly, *dppB*, *dppD*, and *dppF* all belong to the same operon, involved in peptide transport (33), and *dppA*, the first gene in that operon, was present among the priors. In total, 13 novel predictions for GcvB were experimentally supported, including *nlpA*, which was present in five of the six inferred GcvB regulons, and validated as a target in reference 32. Three novel targets, *asd*, *hcxB* (*ybiC*), and *dcyD* were supported by physical binding data (10, 32), as well as a previous report of the GcvB-*dcyD* interaction in *S.* Typhimurium (34). Eleven novel targets lacked direct experimental support but belonged to operons that include known GcvB targets (dotted lines in Fig. 4D).

Figure 4E shows the Spot 42 regulon inferred using as priors 23 CopraRNA-predicted targets with *P* values of ≤0.01 and associated with enriched terms; however, these priors had low experimental support rate (Table S2). Only six priors were recalled, including two (*icd* and *sdhC*) that have been validated and three more with experimental support (Data Set S2). This constitutes another illustration of our method’s ability to select experimentally supported interactions and filter out false-positive interactions. Among the novel targets were *sthA*, a validated Spot 42 target (35), and *mdh*. The Spot 42-*mdh* interaction is supported by physical binding data and consistent with the role of Spot 42 in carbon metabolism (10, 35).

### Expanding the partially characterized sRNA regulons for Spot 42, GcvB, PrrF, FsrA, and RsaE.

**FIG 5:**
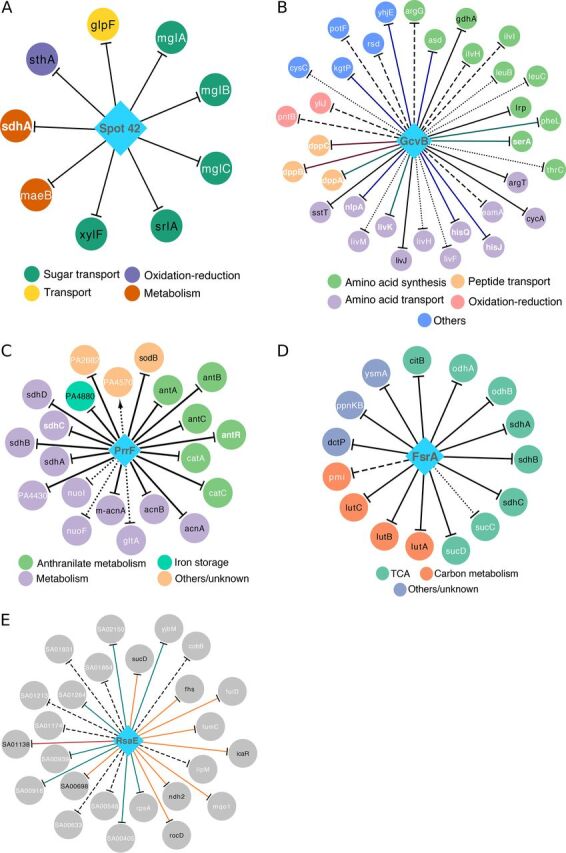
Selected expanded sRNA regulons of E. coli, P. aeruginosa, B. subtilis, and S. aureus. sRNA regulons were inferred using manually selected sRNA priors listed in Table S1. Diamonds and circles represent sRNAs and target genes, respectively. Solid lines indicate priors and experimentally supported novel targets. Dashed lines indicate unsupported predictions. Black node labels indicate prior targets, and white node labels indicate novel targets (not used as sRNA priors). Validated novel targets are shown in bold white font. Target genes are colored according to their functional annotation. (A) The inferred E. coli Spot 42 regulon. All predicted targets were experimentally supported. (B) The inferred E. coli GcvB regulon. Novel interactions supported by transcriptional profiling data, physical binding data, or both are shown in red, blue, and green, respectively. Based on the high number of common GcvB targets in E. coli and *S.* Typhimurium, experimental data from *S.* Typhimurium was considered supporting evidence. Dotted lines indicate targets without direct support but located in the same operon of experimentally supported targets. (C) The inferred PrrF regulon of P. aeruginosa. Dotted lines indicate PrrF targets supported by increased expression at the mRNA or protein levels in high-iron versus low-iron conditions but not in *prrF* deletion strains. (D) The inferred FsrA regulon of B. subtilis. Dotted lines indicate FsrA targets supported by mRNA upregulation in the *fur* deletion strain (with respect to the wild-type strain) but not in the *fsrA fur* double deletion strain (with respect to *fur* deletion strain). (E) The inferred S. aureus RsaE regulon. Experimental support was evaluated using transcriptional profiling data of *rsaE* deletion, *rsaE* overexpression, and limited RsaE-mRNA binding data reported by Rochat et al. (52). Green, orange, and red lines indicate RsaE targets supported by one, two, and three data types, respectively. Due to space constraints, the first part of the locus names (SAOUHSC_) was abbreviated to “SA” in panel E.

For five selected sRNAs, we show how our approach identified 30 novel sRNA-mRNA interactions with experimental support in E. coli, P. aeruginosa, B. subtilis, and S. aureus (Data Set S1). Spot 42 (encoded by *spf*) controls sugar metabolism (35), and Fig. 5A reports its predicted regulon (inferred using experimentally supported priors rather than CopraRNA predictions). Four (out of 12) priors were recalled, and five novel targets were predicted. The first novel target, *maeB*, encodes an NADP-dependent malate dehydrogenase (36). It had not been flagged as differentially expressed in previous experiments (35); however, after reanalysis of available transcriptional data with a Bayesian *t* test (37), we found that *maeB* was significantly downregulated in response to *spf* overexpression (−2.12 average fold change). Since a physical interaction between Spot 42 and *maeB* mRNA was recently reported (10), we conclude that *maeB* is a genuine Spot 42 target. Because the NAD-dependent malate dehydrogenase encoded by *maeA* (an independent transcriptional unit from *maeB*) is a known Spot 42 target (35, 36), interaction of Spot 42 with both *mae* transcripts would likely result in a complete block of pyruvate synthesis from malate. Another novel predicted target was *mglB*, which encodes a galactose ABC transporter (38). This gene was significantly downregulated in an *spf*-overexpressing strain (−2.69 average fold change) and was predicted as a target by CopraRNA (Fig. 4E). Its interaction with Spot 42 was recently validated in *S.* Typhimurium (39). We conclude that the *mgl* operon is also a true target in E. coli, implying that Spot 42 represses galactose metabolism and transport through repression of both the *gal* (40) and *mgl* operons. The last novel target was *sdhA*, in agreement with a previously reported interaction between Spot 42 and *sdhC*, another member of the *sdh* operon (41). Desnoyers and Massé (41) found that Spot 42 primarily regulates *sdh* at the translational level. Yet, our approach was able to detect this interaction, maybe because it is sensitive to subtle changes in mRNA stability (degradation of the *sdh* mRNA was observed 30 min after *spf* induction) or because Spot 42 promotes faster degradation of the *sdh* polycistronic transcript in a still unidentified condition. Overall, all five of the Spot 42 novel targets were experimentally supported.

GcvB is known to regulate amino acid biosynthesis and transport (32). An expanded E. coli GcvB regulon (inferred from the compiled set of experimentally supported sRNA priors) is shown in Fig. 5B. Six (out of nine) priors were recovered as GcvB targets, and 27 additional targets were predicted. Of these novel targets, 8 have been independently validated (shown in bold white font in Fig. 5B; Data Set S1), and 10 had support from physical binding data (hypergeometric test *P* value of <1e−07) (10). Notably, six novel targets (*asd*, *kgtP*, *hisJ*, *hisQ*, *nlpA*, and *yhjE*), although not supported by transcriptomic data, were detected as physically interacting with GcvB *in vivo* (10, 32). Two additional predictions (*thrC* and *cysC*) were indirectly supported. The leader sequence of the *thr* operon, *thrL*, interacts with GcvB and is a confirmed GcvB target in *S.* Typhimurium (10, 34), whereas GcvB physically interacts with the transcript of a transcriptional regulator of *cysC*, CysB (10). The GcvB-*asd* interaction appears to be conserved among multiple species, since higher Asd levels were observed in *gcvB* and *hfq* deletion strains of Pasteurella multocida (42). A challenge for new technologies assessing binding of sRNA to mRNAs, such as RNA interaction by ligation and sequencing (RIL-Seq) (10), *in vivo* UV cross-linking with RNA deep sequencing (39), and MS2 affinity purification coupled with RNA sequencing (32), is to identify whether the detected interactions actually influence mRNA stability and/or translation rate (10, 43). Our approach constitutes a complementary tool to identify which interactions have functional relevance among the hundreds of detected binding events.

PrrF1 and PrrF2 in P. aeruginosa are functional analogs of E. coli RyhB (44) and B. subtilis FsrA (45). Both PrrFs are transcriptionally repressed by Fur under iron-rich conditions (44). Since PrrF1 and PrrF2 are almost identical at the sequence level (44), they were considered a single regulator (PrrF) in our analysis. The predicted PrrF regulon included all 11 priors and 10 novel targets (Fig. 5C and Data Set S1). Among the novel predictions, two (*antR* and *sdhC*) are experimentally validated targets (46, 47), and three (PA2682, *catA*, and *catC*) were significantly upregulated in P. aeruginosa cells grown under high- versus low-iron conditions, as well as in the *prrF1*-*prrF2* deletion mutant versus wild-type (WT) cells (44, 46). PA2682 was also downregulated at the protein level in low- versus high-iron conditions (48). Similarly, PA4430 and PA4570 were supported by the upregulation at the protein level in the *prrF1*-*prrF2* deletion mutant (48) and for PA4430 in high- versus low-iron conditions (48). Unexpectedly, PA4570 protein level was downregulated in high- versus low-iron conditions (48). This may explain a predicted positive interaction between PrrF and PA4570 (Fig. 5C). Furthermore, PA4570 could be regulated by both Fur and PrrF, as targets shared by Fur and RyhB have been proposed for E. coli (31). PA4430 and the *catACB* operon were also predicted as PrrF targets by CopraRNA (Data Set S1). The predicted PrrF-*gltA* interaction is supported by significant upregulation of *gltA* at both the transcriptional and translational levels in high- versus low-iron conditions (46, 48). Moreover, in B. subtilis, *gltA* is a known target of FsrA (49). Interactions with the *nuo* operon members, *nuoF* and *nuoI*, were supported by the decrease in NuoF and NuoI protein levels in low- versus high-iron conditions (48). Similarly, expression of *nuoA* was induced after iron addition (50). Like *acnA*, *acnB*, and the *sdh* operon, the *nuo* operon may be a target shared by PrrF and RyhB. In summary, all 10 novel PrrF targets predicted by the model are experimentally supported.

Figure 5D shows the inferred FsrA regulon in B. subtilis (45), which recalls 8 (out of 12) priors and predicts seven novel target genes (including six that were experimentally supported). Three novel targets (*odhA*, *odhB*, and *pmi*) are also among the FsrA targets predicted by CopraRNA (Data Set S1). The *odh* operon encodes enzymes involved in the TCA cycle (51), and *odh* transcripts were upregulated (mean fold changes, 1.7 for *odhA* and 1.9 for *odhB*) in the *fsrA*-*fur* deletion mutant compared to the *fur* deletion mutant (49). Similarly, interactions with *ysmA*, *sucD* (of the *sucCD* operon), or *ppnKB* were supported by upregulation under the same conditions (mean fold changes, 3. 8, 2.5, and 11.0, respectively) (49). Furthermore, *sucC* was downregulated (0.5 mean fold change) in the *fur* single deletion mutant compared to the WT (49). Interactions between FsrA and the *odhA*-*odhB* or *sucC*-*sucD* transcripts are particularly promising due to the role played by these genes in the TCA cycle, as already noted for the *sdh* operon, which is a previously known target (45, 49). In addition, *sucC* and *ppnKB* are validated targets of RoxS, another *trans*-encoded sRNA of B. subtilis (17). This may reveal a functional connection between RoxS and FsrA, as implied by the observation that multiple genes regulated by Fur, the transcriptional repressor of FsrA, appear to also be influenced by RoxS (17). Follow-up experiments are required to obtain a definitive answer.

RsaE is a partially characterized sRNA of S. aureus involved in the regulation of arginine degradation (52). Rochat et al. detected more than 300 potential RsaE targets using transcriptomics (deletion and overexpression of *rsaE*) and *in vitro* trapping of sRNA targets (52). We inferred the RsaE regulon (Fig. 5E) using a mix of 10 validated and experimentally supported RsaE targets as priors (Table S1). Seven priors were recalled (five can be considered validated [Data Set S1]), and 18 novel targets were predicted. Of these, three (*folD*, *fumC*, and *mqo1*) were significantly upregulated in the *rsaE* deletion strain and downregulated in an overexpression strain (versus WT). One target (SAOUHSC_00405) was significantly upregulated only in the *rsaE* deletion strain. Six novel targets (*rpsA*, SAOUHSC_00918, SAOUHSC_00939, SAOUHSC_01264, SAOUHSC_02150, and *yjbM*) were significantly downregulated in a *rsaE* overexpression strain. The set of novel targets was thus significantly enriched with genes downregulated in the *rsaE* overexpression strain (hypergeometric test *P* value of <1e−04). In summary, the inferred RsaE regulon includes new candidates and some of the RsaE targets discovered in reference 52.

### Uncovering the most relevant physiological contexts for sRNA-mediated regulation.

Estimated SRA profiles can be directly used to determine the physiological context associated with highest sRNA activity. For any sRNA of interest, conditions of highest SRA can be compared to reveal commonalities. As an example, we analyzed the activity profiles of PrrF in P. aeruginosa and RsaE in S. aureus.

**FIG 6:**
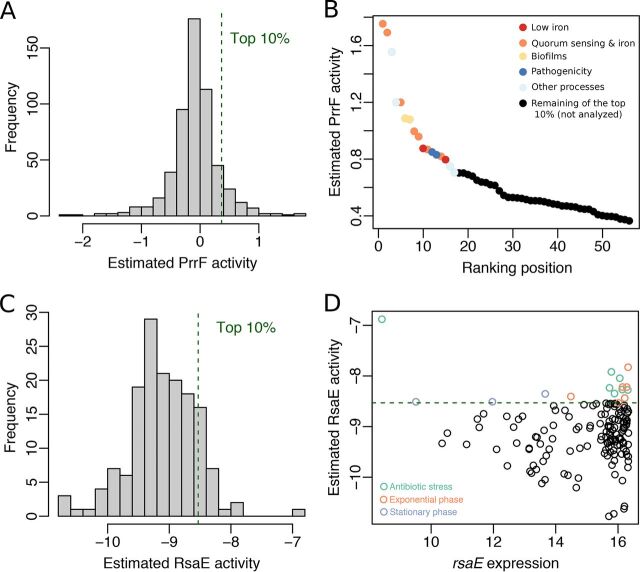
Analysis of sRNA activity profiles reveals the conditions where sRNAs are most likely to interact with their predicted targets. (A) Distribution of the estimated PrrF activity in the 559 experiments included in the P. aeruginosa transcriptomic compendium. (B) Experimental conditions (10% of 559) where PrrF is most active. Each circle depicts the value for one experiment (normalized with respect to a control condition) (60). The first 17 experiments in the ranking are colored according to the corresponding growth conditions. (C) Distribution of the estimated RsaE activity in the 156 experiments included in the S. aureus transcriptomic compendium. (D) Comparison of the complete RsaE activity and expression profiles. Each circle depicts the value for one experiment. Experiments in the top 10% of RsaE activity are colored according to the corresponding growth conditions.

The distribution of estimated PrrF activities is shown in Fig. 6A. We focused on a subset of 56 experiments (out of 559) that corresponds to the 10% of highest PrrF activity. Figure 6B reveals that in the selected experiments, color coded based on the available metadata, PrrF activity follows an exponential distribution. As expected from PrrF’s involvement in the iron-sparing response, this subset included two experiments performed under low-iron conditions (red circles) (Data Set S3). Seven experiments were associated with quorum sensing, i.e., the presence of the *Pseudomonas* quinolone signal (PQS) in the growth medium (orange circles). This observation is explained by the iron chelator properties of PQS (53). Moreover, PrrF is known to connect quorum sensing to iron metabolism (46). PrrF activity was also high in biofilms versus planktonic cultures (yellow circles), consistent with the influence of iron availability on biofilm formation (54). Finally, our results imply that PrrF influences P. aeruginosa pathogenicity. PrrF activities were higher in the presence of neutrophils versus LB medium and in a transmissible P. aeruginosa strain (55) versus the PAO1 strain (blue circles). This hypothesis is supported by the reduced virulence of a PrrF deletion strain (56).

10.1128/mSystems.00057-20.9 DATA SET S3 Estimated sRNA activity profiles of all sRNAs considered in this study. Download Data Set S3, XLSX file, 0.1 MB. Copyright © 2020 Arrieta-Ortiz et al. 2020 Arrieta-Ortiz et al. This content is distributed under the terms of the Creative Commons Attribution 4.0 International license.

We then analyzed the distribution of estimated RsaE activities in S. aureus to uncover the most relevant physiological contexts for this sRNA (Fig. 6C). Analysis of conditions with the 10% of highest RsaE activity (16 experiments) uncovered a link to antibiotic-induced stress responses (linezolid, clindamycin, ciprofloxacin, and colistin challenges). As previously reported (57), RsaE activity was high during exponential phase (Fig. 6D and Data Set S3). RsaE activity and expression profiles showed limited correlation (Spearman’s correlation = 0.127; *P* value = 0.113). This discrepancy is epitomized by the fact that the experiment with the highest RsaE activity (linezolid-treated S. aureus in stationary phase) had the lowest RsaE RNA level in the transcriptomic compendium (Fig. 6D). This observation could be a possible consequence of codegradation of RsaE with its targets mediated by RNase III (58). In summary, analysis of the estimated SRA profiles unveils conditions where sRNAs are most likely to interact with their targets. This knowledge is highly valuable for validating predictions of sRNA-mediated regulation.

### Conclusions.

We have developed a new computational pipeline for inferring bacterial sRNA regulons through integration of TFA and SRA estimates with a reliable network model selection procedure. This strategy is motivated by the realization that using transcriptional profiles of sRNAs as proxies for their activity is not optimal because SRAs are influenced by RNA chaperones, ribonucleases, and sRNA/target ratios (18, 19). Our findings demonstrate that the need to estimate activity of regulatory noncoding RNAs involved in multiple interactions or requiring substantial processing is not exclusive to eukaryotic systems (59).

Our results indicate that integration of SRAs in network inference pipelines significantly improves prediction power in a way that outperforms previous efforts (Fig. 3A). This approach complements sRNA-mRNA prediction methods based on sequence analyses and technologies for detecting physical interactions between sRNAs and mRNAs (Fig. 4). In this work, we report a total of 42 validated and 79 experimentally supported sRNA-mRNA interactions in E. coli, P. aeruginosa, B. subtilis, and S. aureus. These numbers exclude the 64 manually selected sRNA priors recovered in the inferred models and take into account the overlap between the models built with CopraRNA-derived and manually selected sRNA priors (Data Set S1 and Data Set S2). Our strategy is especially well suited for removing the many false-positive interactions introduced in sequence-based predictions of sRNA-mRNA interactions. Hence, it can both expand current sRNA regulons and serve as a guide to study uncharacterized sRNAs.

Our study increased by 101% the number of sRNA priors originally compiled for estimating E. coli SRAs (Table 1). The new set includes 31 independently validated interactions and 45 novel experimentally supported targets (Table S3). Specifically, the contribution of sRNA-mediated regulation in chemotaxis and oxidation-reduction pathways is extended (Fig. 4 and Fig. 5). We also described how a single sRNA (Spot 42) can repress all branches of a metabolic reaction (conversion of malate to pyruvate) and repress the consumption of alternative sugars like galactose by simultaneously inhibiting catabolism and intake. Our approach highlights the functional role of bacterial sRNAs as fine tuners of gene expression.

10.1128/mSystems.00057-20.5 TABLE S3 Experimentally supported new members of the E. coli sRNA regulons inferred in this study. Download Table S3, DOCX file, 0.03 MB. Copyright © 2020 Arrieta-Ortiz et al. 2020 Arrieta-Ortiz et al. This content is distributed under the terms of the Creative Commons Attribution 4.0 International license.

The main limiting factor in our strategy is the requisite for prior information (in the form of a transcriptomic data set, a TF-based transcriptional network, and a list of candidate sRNAs and putative targets). We selected bacterial species for which we could comprehensively assess the quality of the inferred models; however, there is a much larger group of species, including *S.* Typhimurium and Mycobacterium tuberculosis, that would benefit from application of this approach. The COLOMBOS database is a repository of transcriptional compendia for approximately 20 bacterial species (60), and experimentally supported transcriptional interactions can be readily obtained by mining literature and databases, such as RegPrecise (27). In addition, sets of sRNA priors can be generated from sequence-based prediction tools (e.g., CopraRNA), experiments measuring changes in expression levels after inhibition or overexpression of regulators of interest, and global approaches detecting binding of sRNAs to their targets.

The applicability of our strategy will increase as the field of bacterial sRNA-mediated regulation grows. Incorporating estimated TFAs in network inference strategies has led to recent improvements in yeast (61), *Drosophila* (62), and transcriptional networks associated with cancer (63, 64) and differentiation of mouse T lymphocytes (65). We expect that performance will keep improving as the number of confirmed sRNA-mRNA interactions continues to rise and thus the ability to accurately estimate SRAs.

## MATERIALS AND METHODS

### Bacterial species.

We inferred transcriptional regulatory networks and small noncoding RNA regulons for E. coli, P. aeruginosa, S. aureus, and B. subtilis.

### Small noncoding RNA priors.

sRNA-mRNA interactions used as sRNA priors for SRA estimation in each species are listed in Table S1 in the supplemental material. To avoid overrepresentation of any operon in manually selected sRNA priors for E. coli, only one member of each operon containing multiple validated sRNA targets was considered. Priors for RsaE, PrrFs, and FsrA were obtained from reference 52, references 44, 46, and 66, and references 45, 49, and 67, respectively.

### Transcriptomic data sets.

The transcriptomic data sets used for inferring the transcriptional and sRNA networks are described in Table S4.

10.1128/mSystems.00057-20.6 TABLE S4 Transcriptional prior networks and transcriptomic data sets used in this study. Download Table S4, DOCX file, 0.03 MB. Copyright © 2020 Arrieta-Ortiz et al. 2020 Arrieta-Ortiz et al. This content is distributed under the terms of the Creative Commons Attribution 4.0 International license.

### Prior transcriptional networks.

For each species, the prior transcriptional network was constructed as a collection of experimentally supported, signed (activation or repression), TF-gene interactions. The prior networks were used for estimating the regulatory activities of TFs included as potential regulators, inferring the corresponding transcriptional network, and defining the final model of the inferred networks (see below). Sources for each species are shown in Table S4.

### Estimation of transcription factor and sRNA regulatory activities.

TFAs and SRAs were simultaneously estimated using the set of experimentally supported interactions in the prior network as described in reference 24. Briefly, we first combined the sRNA and transcriptional prior networks into a global prior network. We represented the analyzed transcriptional data set in matrix format (referred to as ***X***) where each row corresponded to the transcriptional profile of a gene. Then, we applied a network component analysis (NCA) (68) to decompose ***X*** in two matrices: a first matrix ***P***, which we derived from the prior network. The values in ***P*** are in the {0, 1, −1} set, where 1 and −1 indicate activation and repression, respectively. The value in the *P_i_*_,_*_j_* entry corresponds to the interaction between gene *i* and regulator *j*. The second matrix ***A*** is unknown but represents the activities of regulators along the conditions in ***X***. As such, the *A_k_*_,_*_l_* entry is the activity of regulator *k* in condition *l*. In matrix notation, NCA can be stated as:

(1) X=PA

We solved for ***A*** using the pseudoinverse of ***P*** as explained in reference 24.

### Inference of transcriptional and sRNA networks.

TF and sRNA networks were simultaneously inferred using *Inferelator* Bayesian best subset regression (BBSR), as detailed in reference 24. The core model of the *Inferelator* with incorporation of TFAs and SRAs can be summarized as:

(2) $$X_{i,j}=\underset{k\in \{\text{TFs U sRNAs}\}}{\sum }\beta_{i,k}{Â}_{k,j}$$

where *X_i_*_,_*_j_* is the mRNA level of gene *i* in condition *j*, *Â* is the matrix of estimated activities generated with NCA (as described above), and β*_i_*_,_*_k_* indicates the effect (positive or negative) and strength of regulator *k*’s activity on gene *i*. β is the main output of the *Inferelator*. To model the sparsity of biological networks, BBSR solves for a matrix β where most values are zero. More details about BBSR solution can be found in reference 24. To avoid overfitting, we bootstrapped the input transcriptional data 20 times (we have previously observed minimal change above 20 bootstraps) (24). We averaged the β scores associated with each resampling instance into a final β matrix. The second output of the *Inferelator* is a confidence score matrix generated as explained in reference 24. The confidence score of an interaction indicates the likelihood of the interaction. Mixed CLR was run using the *mi_and_clr.R* script in the *Inferelator* release, available at https://sites.google.com/a/nyu.edu/inferelator/home.

### Construction of final model of TF and sRNA networks.

We ranked the set of all potential regulator (TF/sRNA)-gene interactions based on the associated confidence scores. We used a 0.5 precision cutoff (as previously used in reference 24) to define the set of interactions included in the final model. The confidence cutoff was defined as the score at which exactly 50% of the TF-gene and sRNA-gene interactions above the cutoff were part of the prior network.

### Validation of inferred sRNA regulons.

To assess the accuracy of the inferred sRNA regulons, we mined publicly available transcriptional profiling data sets, sRNA-mRNA binding data, and results of other relevant experiments (Northern blots, point mutations, translational fusions, ribosome profiling, *in silico* predictions, etc.) for each species. A total of 383 candidate E. coli sRNA-mRNA interactions were retrieved from available literature (excluding binding data). This set of potential interactions was extended to 689 to include genes located in the same operons, as predicted in MicrobesOnline (69). Independent studies supporting novel sRNA-mRNA interactions discussed in the text are cited in the relevant sections.

### sRNA prior shuffling and noisy sRNA priors.

To estimate the basal performance of the *Inferelator* algorithm when SRA was incorporated, sRNA priors were randomly shuffled while conserving the original size of each sRNA regulon. To add different amounts of false interactions to the manually curated E. coli sRNA priors, additional targets were randomly selected from the genes not supported as potential sRNA targets (i.e., located outside operons that contain genes differentially expressed in transcriptional profiling experiments perturbing sRNA expression or other validated sRNA targets).

### Differential expression analysis of *spf* overexpression in E. coli microarray data.

To evaluate whether additional candidate targets of Spot 42 could be identified, differential expression analysis was performed, using a Bayesian T-test with Cyber-T (37). We analyzed the normalized microarray data of *spf* overexpression (GEO accession no. GSE24875) reported in reference 35. To consider a gene differentially expressed, Beisel and Storz used a minimum twofold change in each of the replicates and only 16 genes fit that criterion (35). In our analysis, we considered only genes included in the E. coli transcriptomic data used as input for the *Inferelator* and excluded genes that were absent in any of the replicates. Genes with *P* values of ≤0.01 were considered differentially expressed. Adjusted *P* values were not used because only five and seven genes had adjusted *P* values of ≤0.05 or adjusted *P* values of ≤0.1, respectively. This would narrow down the set of potential targets (not our goal), and it would leave out previously validated Spot 42 targets. We have successfully used a 0.01 raw *P* value threshold for analyzing B. subtilis transcriptomic data (24). Thirteen out of the 16 genes originally labeled as differentially expressed in reference 35 were recovered, and 25 additional differentially expressed genes were detected.

### *In silico* prediction of sRNA-mRNA interactions.

For sRNAs that were conserved among multiple bacterial species, precomputed predictions from the CopraRNA website (http://rna.informatik.uni-freiburg.de/CopraRNA/Input.jsp) were downloaded and used as priors. When predictions were not available for an sRNA of interest, a new run was submitted to the CopraRNA website. All CopraRNA predictions for E. coli sRNAs were downloaded between January and July 2016.

### Analysis of publicly available data for physical sRNA-mRNA interactions detected by MS2 affinity purification coupled with RNA sequencing.

Lalaouna and collaborators have successfully developed and applied the MS2 affinity purification coupled with RNA sequencing (MAPS) technology to detect mRNAs that physically interact with RyhB and GcvB sRNAs (32, 70). For each potential interaction detected with MAPS, its frequency in a bacterial strain expressing the sRNA of interest with an MS2 tag and a control strain is compared. In this way, interacting RNA-RNA pairs with higher ratio values are expected to have a higher probability of being true physical interactions. We downloaded the ratio scores for the RyhB and GcvB interactomes reported in references 32 and 70. Because those data sets contained all detected interactions (4,362 and 7,374 for RyhB and GcvB, respectively) and there is no standard ratio threshold to distinguish true from spurious binding events, we used a ratio of 20 as our threshold to consider an sRNA-mRNA interaction as supported by MAPS. We chose this stringent threshold (there are validated RyhB and GcvB targets with ratios smaller than 20) based on the fact that it was the lowest value among the newly validated sRNA targets reported in references 32 and 70. Using this threshold, only 5.5% and 7% of the potential RyhB and GcvB interactome were considered supported by MAPS. To take into account random binding between RNAs and MS2, we considered only interactions that have at least twice the ratio score in the strain expressing the MS2-tagged sRNA with respect to the ratio reported for a strain expressing MS2 alone (43).

### Network visualization.

sRNA networks and regulons were visualized using Cytoscape v 3.4.0 (71).

### Code availability.

R scripts and necessary input files to infer the described sRNA regulons are publicly available in the following GitHub repository: https://github.com/marioluisao/sRNA_networks.
