# Supplementary material for: Inference of Bacterial Small RNA Regulatory Networks and Integration with Transcription Factor-Driven Regulatory Networks
Source: mSystems. 2020 Jun 2;5(3):e00057-20. doi: 10.1128/mSystems.00057-20 (PMC8534726; doi:10.1128/mSystems.00057-20)
Supplement: TABLE S1 [file msystems.00057-20-st001.docx]

| **Table S1. Manually selected sRNA-mRNA interactions used as sRNA priors in this study** | | | | |
| --- | --- | --- | --- | --- |
| **Species** | **sRNA** | **Target gene** | **Locus** |  |
| *E. coli* | CyaR | *ompX* | b0814 |  |
| *E. coli* | CyaR | *nadE* | b1740 |  |
| *E. coli* | CyaR | *yobF* | b1824 |  |
| *E. coli* | CyaR | *ptsI* | b2416 |  |
| *E. coli* | CyaR | *yqaE* | b2666 |  |
| *E. coli* | CyaR | *luxS* | b2687 |  |
| *E. coli* | FnrS | *gpmA* | b0755 |  |
| *E. coli* | FnrS | *cydD* | b0887 |  |
| *E. coli* | FnrS | *maeA* | b1479 |  |
| *E. coli* | FnrS | *marA* | b1531 |  |
| *E. coli* | FnrS | *sodB* | b1656 |  |
| *E. coli* | FnrS | *yobA* | b1841 |  |
| *E. coli* | FnrS | *folE* | b2153 |  |
| *E. coli* | FnrS | *folX* | b2303 |  |
| *E. coli* | FnrS | *metE* | b3829 |  |
| *E. coli* | FnrS | *sodA* | b3908 |  |
| *E. coli* | GcvB | *lrp* | b0889 |  |
| *E. coli* | GcvB | *csgD* | b1040 |  |
| *E. coli* | GcvB | *phoP* | b1130 |  |
| *E. coli* | GcvB | *gdhA* | b1761 |  |
| *E. coli* | GcvB | *argT* | b2310 |  |
| *E. coli* | GcvB | *sstT* | b3089 |  |
| *E. coli* | GcvB | *livJ* | b3460 |  |
| *E. coli* | GcvB | *yifK* | b3795 |  |
| *E. coli* | GcvB | *cycA* | b4208 |  |
| *E. coli* | MicA | *tsx* | b0411 |  |
| *E. coli* | MicA | *ompX* | b0814 |  |
| *E. coli* | MicA | *ompA* | b0957 |  |
| *E. coli* | MicA | *ycfS* | b1113 |  |
| *E. coli* | MicA | *ompW* | b1256 |  |
| *E. coli* | MicA | *lpxT* | b2174 |  |
| *E. coli* | MicA | *yfeK* | b2419 |  |
| *E. coli* | MicA | *lamB* | b4036 |  |
| *E. coli* | MicA | *fimB* | b4312 |  |
| *E. coli* | MicA | *ecnB* | b4411 |  |
| *E. coli* | OmrA | *ompT* | b0565 |  |
| *E. coli* | OmrA | *fepA* | b0584 |  |
| *E. coli* | OmrA | *csgD* | b1040 |  |
| *E. coli* | OmrA | *cirA* | b2155 |  |
| *E. coli* | OmrA | *ompR* | b3405 |  |
| *E. coli* | OmrA | *fecA* | b4291 |  |
| *E. coli* | RybB | *tsx* | b0411 |  |
| *E. coli* | RybB | *nmpC* | b0553 |  |
| *E. coli* | RybB | *fiu* | b0805 |  |
| *E. coli* | RybB | *ompF* | b0929 |  |
| *E. coli* | RybB | *ompA* | b0957 |  |
| *E. coli* | RybB | *ompW* | b1256 |  |
| *E. coli* | RybB | *ompC* | b2215 |  |
| *E. coli* | RybB | *fadL* | b2344 |  |
| *E. coli* | RybB | *rluD* | b2594 |  |
| *E. coli* | RybB | *lamB* | b4036 |  |
| *E. coli* | RyhB | *erpA* | b0156 |  |
| *E. coli* | RyhB | *ykgJ* | b0288 |  |
| *E. coli* | RyhB | *dmsA* | b0894 |  |
| *E. coli* | RyhB | *nagZ* | b1107 |  |
| *E. coli* | RyhB | *acnA* | b1276 |  |
| *E. coli* | RyhB | *ynfF* | b1588 |  |
| *E. coli* | RyhB | *fumA* | b1612 |  |
| *E. coli* | RyhB | *sodB* | b1656 |  |
| *E. coli* | RyhB | *msrB* | b1778 |  |
| *E. coli* | RyhB | *cysE* | b3607 |  |
| *E. coli* | RyhB | *fumB* | b4122 |  |
| *E. coli* | RyhB | *uof* | b4637 |  |
| *E. coli* | Spot 42 | *gltA* | b0720 |  |
| *E. coli* | Spot 42 | *galK* | b0757 |  |
| *E. coli* | Spot 42 | *puuE* | b1302 |  |
| *E. coli* | Spot 42 | *paaK* | b1398 |  |
| *E. coli* | Spot 42 | *maeA* | b1479 |  |
| *E. coli* | Spot 42 | *srlA* | b2702 |  |
| *E. coli* | Spot 42 | *ascF* | b2715 |  |
| *E. coli* | Spot 42 | *fucI* | b2802 |  |
| *E. coli* | Spot 42 | *xylF* | b3566 |  |
| *E. coli* | Spot 42 | *glpF* | b3927 |  |
| *E. coli* | Spot 42 | *sthA* | b3962 |  |
| *E. coli* | Spot 42 | *nanC* | b4311 |  |
| *P. aeruginosa* | PrrF | *m-acnA* | PA0794 |  |
| *P. aeruginosa* | PrrF | *acnA* | PA1562 |  |
| *P. aeruginosa* | PrrF | *sdhD* | PA1582 |  |
| *P. aeruginosa* | PrrF | *sdhA* | PA1583 |  |
| *P. aeruginosa* | PrrF | *sdhB* | PA1584 |  |
| *P. aeruginosa* | PrrF | *acnB* | PA1787 |  |
| *P. aeruginosa* | PrrF | *antA* | PA2512 |  |
| *P. aeruginosa* | PrrF | *antB* | PA2513 |  |
| *P. aeruginosa* | PrrF | *antC* | PA2514 |  |
| *P. aeruginosa* | PrrF | *sodB* | PA4366 |  |
| *P. aeruginosa* | PrrF | - | PA4880 |  |
| *B. subtilis* | FsrA | *dctP* | BSU04470 |  |
| *B. subtilis* | FsrA | *citB* | BSU18000 |  |
| *B. subtilis* | FsrA | *gltB* | BSU18440 |  |
| *B. subtilis* | FsrA | *gltA* | BSU18450 |  |
| *B. subtilis* | FsrA | *leuD* | BSU28250 |  |
| *B. subtilis* | FsrA | *leuC* | BSU28260 |  |
| *B. subtilis* | FsrA | *sdhB* | BSU28430 |  |
| *B. subtilis* | FsrA | *sdhA* | BSU28440 |  |
| *B. subtilis* | FsrA | *sdhC* | BSU28450 |  |
| *B. subtilis* | FsrA | *lutC* | BSU34030 |  |
| *B. subtilis* | FsrA | *lutB* | BSU34040 |  |
| *B. subtilis* | FsrA | *lutA* | BSU34050 |  |
| *S. aureus* | RsaE | - | SAOUHSC_00698 |  |
| *S. aureus* | RsaE | *ndh2* | SAOUHSC_00875 |  |
| *S. aureus* | RsaE | *rocD* | SAOUHSC_00894 |  |
| *S. aureus* | RsaE | - | SAOUHSC_00951 |  |
| *S. aureus* | RsaE | - | SAOUHSC_01138 |  |
| *S. aureus* | RsaE | *sucD* | SAOUHSC_01218 |  |
| *S. aureus* | RsaE | *gcvT* | SAOUHSC_01634 |  |
| *S. aureus* | RsaE | *fhs* | SAOUHSC_01845 |  |
| *S. aureus* | RsaE | *rocF* | SAOUHSC_02409 |  |
| *S. aureus* | RsaE | *icaR* | SAOUHSC_03001 |  |
