# Supplementary figures and images for: Inference of Bacterial Small RNA Regulatory Networks and Integration with Transcription Factor-Driven Regulatory Networks
Source: mSystems. 2020 Jun 2;5(3):e00057-20. doi: 10.1128/mSystems.00057-20 (PMC8534726; doi:10.1128/mSystems.00057-20)

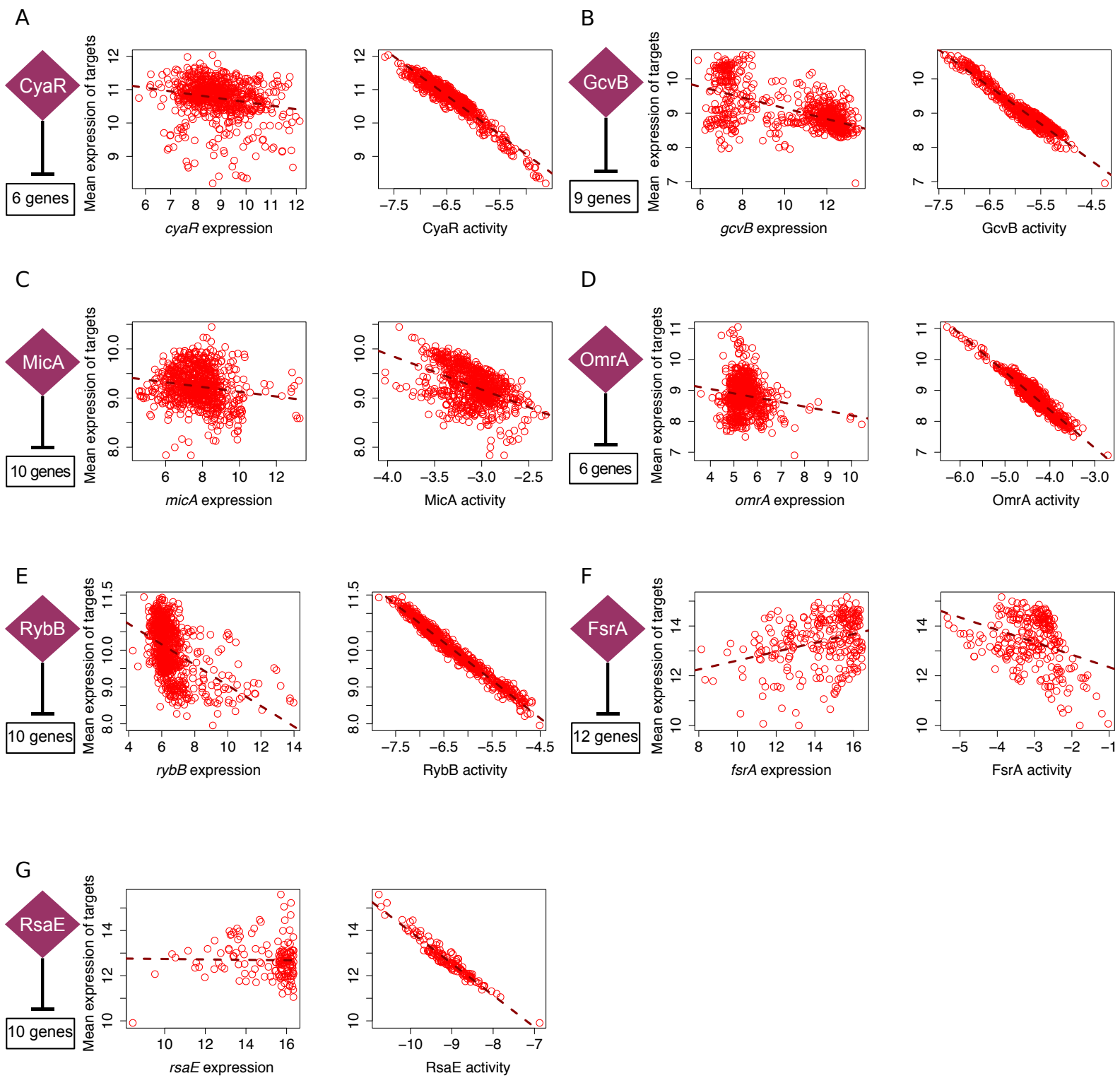

Supplement: FIG S1 [file msystems.00057-20-sf001.pdf]

A

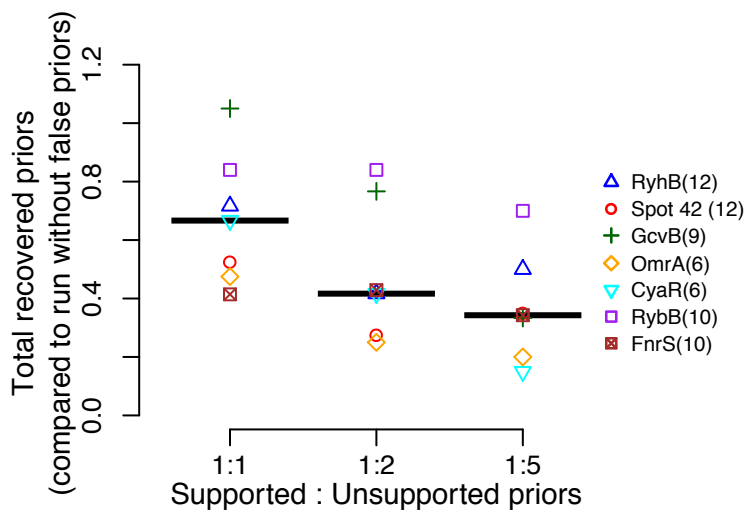

B

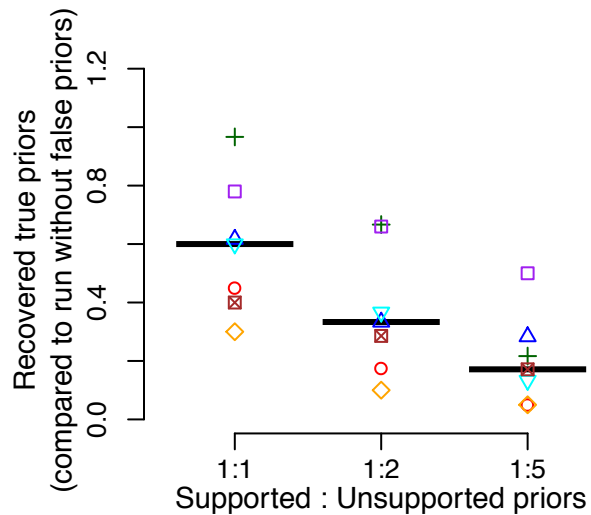

Supplement: FIG S2 [file msystems.00057-20-sf002.pdf]
