# Supplementary material for: Inference of Bacterial Small RNA Regulatory Networks and Integration with Transcription Factor-Driven Regulatory Networks
Source: mSystems. 2020 Jun 2;5(3):e00057-20. doi: 10.1128/mSystems.00057-20 (PMC8534726; doi:10.1128/mSystems.00057-20)
Supplement: TABLE S2 [file msystems.00057-20-st002.docx]

**Table S2****.**  **The *Inferelator* filters CopraRNA-derived priors and predicts novel sRNA-mRNA interactions with experimental support.**

| **sRNA priors selection*^a^*** | **sRNA** | **Priors** | **Supported**  **priors*^b^*** | **Priors predicted as targets*^c^*** | **New**  **targets*^c^*** |
| --- | --- | --- | --- | --- | --- |
| Top 100 | RyhB | 100 | 29 (0.29) | 5 (1) | 6 (0.17) |
|  | GcvB | 100 | 30 (0.3) | 3 (0.67) | 12 (0.5) |
|  | Spot 42 | 100 | 17 (0.17) | 6 (0.33) | 27 (0.07) |
| P-values ⩽ 0.01 | RyhB | 49 | 17 (0.35) | 5 (0.4) | 29 (0.17) |
|  | GcvB | 46 | 21 (0.46) | 11 (0.82) | 39 (0.44) |
|  | Spot 42 | 54 | 12 (0.22) | 4 (0.25) | 17 (0.06) |
| Annotated with enriched terms | RyhB | 38 | 19 (0.5) | 6 (1) | 9 (0.44) |
|  | GcvB | 34 | 19 (0.56) | 11 (0.82) | 43 (0.42) |
|  | Spot 42 | 43 | 11 (0.26) | 5 (0.4) | 6 (0.17) |
| P-values ⩽ 0.01 AND annotated with enriched terms | RyhB | 20 | 11 (0.55) | 1 (1) | 6 (0.33) |
|  | GcvB | 22 | 16 (0.73) | 13 (0.77) | 37 (0.49) |
|  | Spot 42 | 23 | 8 (0.35) | 6 (0.67) | 12 (0.17) |
| P-values ⩽ 0.01 OR annotated with enriched terms | RyhB | 67 | 25 (0.37) | 4 (1) | 1 (0) |
|  | GcvB | 58 | 24 (0.41) | 10 (0.8) | 39 (0.36) |
|  | Spot 42 | 74 | 15 (0.2) | 7 (0.29) | 17 (0.06) |
| Top 15+*^d^* | RyhB | 29 | 14 (0.48) | 1 (1) | 8 (0.38) |
|  | GcvB | 25 | 16 (0.64) | 10 (0.8) | 36 (0.53) |
|  | Spot 42 | 32 | 10 (0.31) | 4 (0.5) | 14 (0.14) |

Predicted targets that physically interact with relevant sRNAs according to binding data reported by Melamed et al. (2016), or present in the set of potential sRNA targets defined in this study, using transcriptional profiling data and validated targets (Table 1), were considered supported. Members of operons with differentially expressed genes in sRNA perturbation (or validated targets) were also considered experimentally supported.

*^a^*Each sRNA regulon was inferred using six versions of the CopraRNA-derived priors.

*^b^*Experimental support rate of the full set of priors is shown in parentheses.

*^c^*Experimental support rate is shown in parentheses.

*^d^*The union of the top 15 predictions (ranked by associated p-values), and the set of targets with p-values ⩽ 0.01 and annotated with enriched terms.
