# Supplementary material for: Inference of Bacterial Small RNA Regulatory Networks and Integration with Transcription Factor-Driven Regulatory Networks
Source: mSystems. 2020 Jun 2;5(3):e00057-20. doi: 10.1128/mSystems.00057-20 (PMC8534726; doi:10.1128/mSystems.00057-20)
Supplement: TABLE S3 [file msystems.00057-20-st003.docx]

**Table S3. Experimentally supported new members of the *E. coli* sRNA regulons inferred in this study.**

| **sRNA** | **Target*^a^*** | **Experimental**  **support*^b^*** | **Inferelator Run*^c^*** | **References*^d^*** |
| --- | --- | --- | --- | --- |
| RyhB | *acpP* | B | CopraRNA | (1) |
| RyhB | *amn* | B | Both | (2) |
| RyhB | *cheY* | B, S | CopraRNA | (1, 3) |
| RyhB | *fabZ* | B | CopraRNA | (1, 2) |
| RyhB | *folX* | B | CopraRNA | (1) |
| RyhB | *gshB* | B | CopraRNA | (1) |
| RyhB | *mrp* | TD, B, RP | CopraRNA | (1, 2, 4, 5) |
| RyhB | *narG* | B, RP | Manual | (1, 4) |
| RyhB | *rna* | B | CopraRNA | (2) |
| RyhB | *rsmE* | B | CopraRNA | (2) |
| RyhB | *tpx* | B | CopraRNA | (1) |
| RyhB | *ubiD* | B | CopraRNA | (1) |
| RyhB | *ybaB* | B | CopraRNA | (1) |
| RyhB | *ycfP* | B | Manual | (2) |
| GcvB | *aroG* | B | CopraRNA | (6) |
| GcvB | *aroP* | B, S | CopraRNA | (1, 6, 7) |
| GcvB | *asd* | B | Both | (1) |
| GcvB | *cysD* | TD | CopraRNA | (8) |
| GcvB | *dcyD (yedO)* | B, S | CopraRNA | (1, 7) |
| GcvB | *hcxB* (*ybiC*) | B | CopraRNA | (1, 6) |
| GcvB | *icd* | B, S | CopraRNA | (1, 9) |
| GcvB | *ilvN* | TD, S | CopraRNA | (7, 8) |
| GcvB | *kgtP* | B | Manual | (1, 6) |
| GcvB | *leuA* | B | CopraRNA | (6) |
| GcvB | *pheL* | B, S | Manual | (1, 7) |
| GcvB | *purU* | B, S | CopraRNA | (1, 6, 7) |
| GcvB | *ydiJ* | B,S | CopraRNA | (1, 6, 7) |
| GcvB | *yecS* | S | CopraRNA | (7) |
| GcvB | *yhjE* | B | Manual | (1) |
| Spot 42 | *fabA* | TD*^e^* | CopraRNA | (10) |
| Spot 42 | *fadL* | TD*^e^* | CopraRNA | (10) |
| Spot 42 | *lpd* | B | CopraRNA | (1) |
| Spot 42 | *lysS* | B, I | CopraRNA | (1, 11) |
| Spot 42 | *maeB* | TD*^e^*, B | Manual | (1, 10) |
| Spot 42 | *mdh* | B | CopraRNA | (1) |
| Spot 42 | *mglA* | TD*^e^*, S | Manual | (10, 12) |
| Spot 42 | *mglB* | TD*^e^*, S | Both | (10, 12) |
| Spot 42 | *mglC* | TD*^e^*, S | Manual | (10, 12) |
| Spot 42 | *rbsB* | TD*^e^* | CopraRNA | (10) |
| Spot 42 | *tktA* | B | CopraRNA | (1) |
| Spot 42 | *yjiA* | B, TD | CopraRNA | (1, 10) |
| Spot 42 | *yjjK* | TD*^e^*,I | CopraRNA | (10, 11) |
| CyaR | *crr* | TD | Manual | (13) |
| FnrS | *grxD* | TD, B | Manual | (1, 14) |
| OmrA | *fecB* | TD | Manual | (15) |

Complete description of experimental data supporting listed interactions is available in **Supplementary Dataset 1** and **Supplementary Dataset 2**.

*^a^* Targets considered supported only due to their location in the same operon of validated sRNA targets (for the relevant sRNA) are not shown.

*^b^“TD”* indicates support from transcriptional data. “*B”* indicates support from physical sRNA-mRNA interaction data. “*S*” indicates support from studies in *S.* Typhimurium*.* “*RP*” indicates support from ribosome profiling data. “I” indicates indirect support (e.g. differential expression in *hfq* deletion strain).

*^c^*Predicted targets were identified using CopraRNA-derived sRNA priors, manually selected sRNA priors or both types of sRNA priors.

*^d^*List of references is included at the end of this document.

*^e^*Gene was identified as differentially expressed in our re-analysis of the transcriptional profiling data reported in (10).

References

1. Melamed S, Peer A, Faigenbaum-Romm R, Gatt YE, Reiss N, Bar A, Altuvia Y, Argaman L, Margalit H. 2016. Global Mapping of Small RNA-Target Interactions in Bacteria. Mol Cell 63:884–897.

2. Lalaouna D, Carrier M-C, Semsey S, Brouard J-S, Wang J, Wade JT, Massé E. 2015. A 3′ external transcribed spacer in a tRNA transcript acts as a sponge for small RNAs to prevent transcriptional noise. Mol Cell 58:393–405.

3. Kim JN, Kwon YM. 2013. Identification of target transcripts regulated by small RNA RyhB homologs in *Salmonella*: RyhB-2 regulates motility phenotype. Microbiol Res 168:621–629.

4. Wang J, Rennie W, Liu C, Carmack CS, Prévost K, Caron M-P, Massé E, Ding Y, Wade JT. 2015. Identification of bacterial sRNA regulatory targets using ribosome profiling. Nucleic Acids Res gkv1158.

5. Massé E, Vanderpool CK, Gottesman S. 2005. Effect of RyhB small RNA on global iron use in *Escherichia coli*. J Bacteriol 187:6962–71.

6. Lalaouna D, Eyraud A, Devinck A, Prévost K, Massé E. 2018. GcvB small RNA uses two distinct seed regions to regulate an extensive targetome. Mol Microbiol.

7. Sharma CM, Papenfort K, Pernitzsch SR, Mollenkopf H-J, Hinton JCD, Vogel J. 2011. Pervasive post-transcriptional control of genes involved in amino acid metabolism by the Hfq-dependent GcvB small RNA. Mol Microbiol 81:1144–65.

8. Pulvermacher SC, Stauffer LT, Stauffer G V. 2009. Role of the sRNA GcvB in regulation of *cycA* in *Escherichia coli*. Microbiology 155:106–114.

9. Sittka A, Lucchini S, Papenfort K, Sharma CM, Rolle K, Binnewies TT, Hinton JCD, Vogel J. 2008. Deep sequencing analysis of small noncoding RNA and mRNA targets of the global post-transcriptional regulator, Hfq. PLoS Genet 4:e1000163.

10. Beisel CL, Storz G. 2011. The base-pairing RNA spot 42 participates in a multioutput feedforward loop to help enact catabolite repression in *Escherichia coli*. Mol Cell 41:286–97.

11. Borirak O, Rolfe MD, de Koning LJ, Hoefsloot HCJ, Bekker M, Dekker HL, Roseboom W, Green J, de Koster CG, Hellingwerf KJ. 2015. Time-series analysis of the transcriptome and proteome of *Escherichia coli* upon glucose repression. Biochim Biophys Acta (BBA)-Proteins Proteomics 1854:1269–1279.

12. Holmqvist E, Wright PR, Li L, Bischler T, Barquist L, Reinhardt R, Backofen R, Vogel J. 2016. Global RNA recognition patterns of post-transcriptional regulators Hfq and CsrA revealed by UV crosslinking *in vivo*. EMBO J e201593360.

13. De Lay N, Gottesman S. 2009. The Crp-activated small noncoding regulatory RNA CyaR (RyeE) links nutritional status to group behavior. J Bacteriol 191:461–76.

14. Durand S, Storz G. 2010. Reprogramming of anaerobic metabolism by the FnrS small RNA. Mol Microbiol 75:1215–1231.

15. Guillier M, Gottesman S. 2006. Remodelling of the *Escherichia coli* outer membrane by two small regulatory RNAs. Mol Microbiol 59:231–247.
