# Supplementary material for: Inference of Bacterial Small RNA Regulatory Networks and Integration with Transcription Factor-Driven Regulatory Networks
Source: mSystems. 2020 Jun 2;5(3):e00057-20. doi: 10.1128/mSystems.00057-20 (PMC8534726; doi:10.1128/mSystems.00057-20)
Supplement: TABLE S4 [file msystems.00057-20-st004.docx]

**Table S4. Transcriptional prior networks and transcriptomic datasets used in this study**

| **Species** | **Transcriptional prior network*^a^*** | **Reference*^b^*** | **Transcriptomic dataset*^c^*** | **Reference*^b^*** |
| --- | --- | --- | --- | --- |
| *Escherichia coli* | RegulonDB version 9.0  (1875*^d^*) | (1) | Many Microbe Microarrays*^e^*  (4297 x 861) | (2) |
|  |  |  |  |  |
| *Pseudomonas aeruginosa* | Collection of known transcriptional interactions | (3) | COLOMBOS 3.0*^h^*  (5629 x 559) | (4) |
|  | Experimental sigma factor network | (5) |  |  |
|  | RegPrecise*^f^* (3569*^g^*) | (6) |  |  |
|  |  |  |  |  |
| *Staphylococcus aureus* | SigB regulon | (7) | HG001  (2837 x 156) | (7) |
|  | RegPrecise*^f^*  (798 *^g^*) | (6) |  |  |
| *Bacillus subtilis* | SubtiWiki | (8) | BSB1 (4445 x 269) | (9) |
|  |  |  |  |  |
|  | Transcriptional network constructed with the Inferelator  (2614*^i^*) | (10) |  |  |
|  |  |  |  |  |
| *^a^*Number of interactions in the corresponding network is shown in parentheses.  *^b^*List of references is included at the end of this document. | | | | |
| *^c^*Number of genes and number of arrays in the dataset are shown in parentheses. | | | | |
| *^d^*Only signed interactions with strong or confirmed evidence were included as priors. | | | | |
| *^e^*Version with un-averaged replicates was used. 16 conditions related to sRNA KOs or their regulators were removed. | | | | |
| *^f^*Only signed interactions (activation or repression) were considered. | | | | |
| *^g^*Total number of interactions in the compiled network (including all mentioned sources). | | | | |
| *^h^*Missing fold-change values, distributed among 105 genes, were replaced with zeroes (which is the average fold-change in the expression matrix). | | | | |
| *^i^*Total number of interactions in the compiled network. The network is composed of TF-gene interactions originally reported in SubtiWiki (Michna et al. 2014) that were recovered in the network reconstructed by the *Inferelator*, and experimentally supported novel interactions of the *Inferelator*-reconstructed model (Arrieta-Ortiz, Hafemeister, et al. 2015).  References  1. Gama-Castro S, Salgado H, Santos-Zavaleta A, Ledezma-Tejeida D, Muñiz-Rascado L, García-Sotelo JS, Alquicira-Hernández K, Martínez-Flores I, Pannier L, Castro-Mondragón JA, Medina-Rivera A, Solano-Lira H, Bonavides-Martínez C, Pérez-Rueda E, Alquicira-Hernández S, Porrón-Sotelo L, López-Fuentes A, Hernández-Koutoucheva A, Del Moral-Chávez V, Rinaldi F, Collado-Vides J. 2016. RegulonDB version 9.0: high-level integration of gene regulation, coexpression, motif clustering and beyond. Nucleic Acids Res 44:D133-43.  2. Faith JJ, Driscoll ME, Fusaro VA, Cosgrove EJ, Hayete B, Juhn FS, Schneider SJ, Gardner TS. 2007. Many Microbe Microarrays Database: uniformly normalized Affymetrix compendia with structured experimental metadata. Nucleic Acids Res 36:D866--D870.  3. Galán-Vásquez E, Luna B, Martínez-Antonio A. 2011. The Regulatory Network of *Pseudomonas aeruginosa*. Microb Inform Exp 1:3.  4. Moretto M, Sonego P, Dierckxsens N, Brilli M, Bianco L, Ledezma-Tejeida D, Gama-Castro S, Galardini M, Romualdi C, Laukens K, Collado-Vides J, Meysman P, Engelen K. 2016. COLOMBOS v3.0: leveraging gene expression compendia for cross-species analyses. Nucleic Acids Res 44:D620-3.  5. Schulz S, Eckweiler D, Bielecka A, Nicolai T, Franke R, Dötsch A, Hornischer K, Bruchmann S, Düvel J, Häussler S. 2015. Elucidation of sigma factor-associated networks in *Pseudomonas aeruginosa* reveals a modular architecture with limited and function-specific crosstalk. PLoS Pathog 11:e1004744.  6. Novichkov PS, Kazakov AE, Ravcheev DA, Leyn SA, Kovaleva GY, Sutormin RA, Kazanov MD, Riehl W, Arkin AP, Dubchak I, Rodionov DA. 2013. RegPrecise 3.0 – A resource for genome-scale exploration of transcriptional regulation in bacteria. BMC Genomics 14.  7. Mäder U, Nicolas P, Depke M, Pané-Farré J, Debarbouille M, van der Kooi-Pol MM, Guérin C, Dérozier S, Hiron A, Jarmer H, Leduc A, Michalik S, Reilman E, Schaffer M, Schmidt F, Bessières P, Noirot P, Hecker M, Msadek T, Völker U, van Dijl JM. 2016. *Staphylococcus aureus* Transcriptome Architecture: From Laboratory to Infection-Mimicking Conditions. PLOS Genet 12:e1005962.  8. Michna RH, Commichau FM, Tödter D, Zschiedrich CP, Stülke J. 2014. SubtiWiki-a database for the model organism *Bacillus subtilis* that links pathway, interaction and expression information. Nucleic Acids Res 42:D692-8.  9. Nicolas P, Mäder U, Dervyn E, Rochat T, Leduc A, Pigeonneau N, Bidnenko E, Marchadier E, Hoebeke M, Aymerich S, Becher D, Bisicchia P, Botella E, Delumeau O, Doherty G, Denham EL, Fogg MJ, Fromion V, Goelzer A, Hansen A, Härtig E, Harwood CR, Homuth G, Jarmer H, Jules M, Klipp E, Le Chat L, Lecointe F, Lewis P, Liebermeister W, March A, Mars R a T, Nannapaneni P, Noone D, Pohl S, Rinn B, Rügheimer F, Sappa PK, Samson F, Schaffer M, Schwikowski B, Steil L, Stülke J, Wiegert T, Devine KM, Wilkinson AJ, van Dijl JM, Hecker M, Völker U, Bessières P, Noirot P. 2012. Condition-dependent transcriptome reveals high-level regulatory architecture in *Bacillus subtilis*. Science 335:1103–6.  10. Arrieta-Ortiz ML, Hafemeister C, Bate AR, Chu T, Greenfield A, Shuster B, Barry SN, Gallitto M, Liu B, Kacmarczyk T, Santoriello F, Chen J, Rodrigues CD, Sato T, Rudner DZ, Driks A, Bonneau R, Eichenberger P. 2015. An experimentally supported model of the *Bacillus subtilis* global transcriptional regulatory network. Mol Syst Biol 11:839–839. | | | | |
